# People living with HIV /AIDS (PLWHA) and HIV/AIDS associated oral lesions; a study in Malaysia

**DOI:** 10.1186/1471-2458-12-850

**Published:** 2012-10-08

**Authors:** Saad A Khan, Jayanthy Moorthy, Hanan Omar, Syed Shahzad Hasan

**Affiliations:** 1 International Medical University, Jalan Jalil Perkasa 19, Bukit Jalil, Kuala Lumpur, 57000, Malaysia; 2 School of Pharmacy, The University of Queensland, 20 Cornwall Street,Woolloongabba, Brisbane, 4102, Australia

## Abstract

**Background:**

The continuous increase in number of people living with HIV/AIDS (PLWHA) represents a serious health and economic burden. HIV positive individuals with oral lesions have significantly lower oral health-related quality of life than HIV positive individuals without oral lesions. The objective of this study was to assess the knowledge, attitude and practices (KAP) within a cohort of HIV/AIDS positive patients towards HIV/AIDS associated oral lesions.

**Methods:**

Two hundred seventy patients attending a national referral hospital of infectious disease in Malaysia were recruited for the study. The study involved the administration of a validated interview-based questionnaire designed to elicit knowledge, attitude and practices of these patients towards HIV associated oral lesions. The last part of the questionnaire assessed the training provided to the patients in relation to the oral lesions associated with the disease and the effectiveness of this training. Data analysis was carried out using SPSS version 18.

**Results:**

Thirty seven percent of patients were reported as knowledgeable, while sixty four percent reported to have positive attitude towards the care of oral hygiene. Sixty six percent of the patients reported that they would seek professional care when experiencing oral lesion. Training was reported effective for 93% patients.

**Conclusions:**

Patients were non-knowledgeable in relation to oral manifestations of the disease and one third of the participating patients showed negative attitudes towards oral health care and reported various measures to manage oral lesions rather than seeking professional care. Developing effective educational methodologies can empower patients with knowledge that may translate to positive attitudes and practices.

## Background

HIV/AIDS is considered as a devastating global health problem posing severe challenges in low and middle-income countries. The continuous increase in number of people living with HIV/AIDS (PLWHA) represents a serious health and economic burden that the world is facing. The total number of PLWHA in 2010 was 34 million with an estimated increase of 3.3 million cases annually [1]. In Malaysia, during 2004 the number of individuals infected with HIV and the number of AIDS cases reported were 6,427 and 1,148 respectively and in 2005, 6,120 were infected by HIV and 1,221 of AIDS cases were reported. The number of PLWHA reached a cumulative total of 87,710 by 2009, the most recent figure in 2011 has reached upto a cumulative figure of 79,855 [2]. The large number of PLWHA – although it is slightly decreasing - requires more attention to improve the quality of life of the individuals diagnosed with the disease as HIV/AIDS affects both physical and psychological health [3].

According to Centre of Disease Control (CDC) improving health outcomes of PLWHA is one of the key recommendations proposed in 2010 with an ultimate goal of extending life and improving its quality [4]. Oral health status of PLWHA was identified by WHO as an integral part of optimum case management. CDC recommendations were given to introduce surveillance activities of oral diseases associated with HIV infection in order to ensure appropriate medical evaluation, prevention and treatment [5].

HIV/AIDS may lead to the development of various oral lesions. Several studies have demonstrated that 40–50% of HIV positive individuals have fungal, bacterial or viral infections in oral cavity that are likely to occur early in the course of the disease. These oral lesions have physical, economic, social and psychological consequences on the individuals and subsequent impairment of the oral-health-related-quality of life [6]. Oral lesions that are strongly associated with HIV infections included oral candidiasis, hairy leukoplakia, Kaposi sarcoma, linear gingival erythema, necrotizing ulcerative gingivitis, necrotizing ulcerative periodontitis and non-Hodgkin lymphoma. Dry mouth was reported as one of the oral manifestations experienced by the patients due to decreased salivary flow rate with subsequent increase in the risk of developing dental caries [7]. Introduction of new therapies like High Active Antiretroviral Therapy (HAART) showed a shift in the prevalence of oral lesions manifesting significant decrease in oral candidiasis and hairy leukoplakia with an increase in salivary gland diseases, xerostomia and warts [8]. The impact of HIV/AIDS and oral disease on the Quality of Life (QOL) is well documented involving physical and emotional well being, social support systems, and life roles. It has been reported that HIV positive individuals with oral lesions have significantly lower oral health-related quality of life than HIV positive individuals without oral lesions. The poor quality of life in PLWHA can be attributed to the effect of oral lesions that may alter facial appearance, speech, and cause chewing and swallowing difficulty and pain [9-11]. Poor oral functionality might lead to exacerbation of nutritional problems that may further affect the quality of life [12,13].

Several Knowledge Attitude and practice (KAP) studies have been carried out worldwide in relation to HIV/AIDS transmission and infection, while scanty literature addressed the knowledge, attitude and practices of PLWHA towards HIV/AIDS associated oral lesions [14-17].

The objective of this study was to assess the knowledge, attitude and practices towards HIV/AIDS associated oral lesions within a cohort of HIV/AIDS positive patients. Knowledge identified the level of understanding towards HIV/AIDS associated oral lesions, attitudes addressed their feelings and ideas towards oral manifestations while practices investigated their professional care seeking behavior towards HIV/AIDS associated oral lesions.

## Methods

The study adopted a structured face-to-face interview, based on a questionnaire. PLWHA attending a referral hospital of infectious disease in Malaysia^a^ were approached to participate in the study and interviewed to obtain their knowledge, attitudes and practices related to HIV/AIDS associated oral lesions. Research Ethics approval was sought and obtained from International Medical University – Joint Research and Ethics Committee [B01/08-RES(25)2011]. The study was conducted during a period of 5 months from May 2011 to September 2011.

The study questionnaire was adapted from a study conducted in Johannesburg [18]. A standardized pre-tested questionnaire was used to collect the data. It comprised of three sections; Section A represented the demographics and HIV/AIDS-related training and consisted of 10 items addressing age, gender, marital status, employment status, level of academic education. The last part of this section assessed whether the individual had received an educational training towards living with HIV/AIDS and its effectiveness. Section B was the knowledge-related section which assessed the knowledge towards common oral manifestation associated with HIV/AIDS as classified by the European Clearinghouse Classification (ECC) and WHO (EC-Clearinghouse, 1993) and the care for these lesions. This section consisted of 7 items with a dichotomous response (yes/no). Each item when answered positively was given a score of one if the response is “yes” and score of zero when the response was “no”. The participants were classified into two subgroups based on the score of these items. Patients with scores higher than 4 were classified as knowledgeable (K) and scores equal to or below 4 were classified as non-knowledgeable (NK). Section C addressed the attitudes and beliefs related to oral care in six dichotomous items. Item 1 and 6 represented positive attitudes, when the response was “yes” a score of one was given and score of zero when the response was “no”. Items 2,3,4 and 5 represented negative attitude and when the response was “yes” was given score of zero and when it is “no” it was a score of 1. The participants are classified into two subgroups based on the score of these items. Patients with scores higher than 3 were classified as having positive attitude while patients with scores equal to or below 3 were classified as having negative attitude. Section D consisted of seven dichotomous items (yes/no) represented the practices towards oral manifestations associated with HIV. Responding with “yes” to seeking profesional care in relation to the 7 lesions stated in the knowledge section was given a score of one while responding with “no” was given the score of zero. Patients with scores higher than 3 were classified as those seeking professional care while patients with scores equal or below 3 were classified as individuals not seeking professional care. The content of the questionnaire was piloted among ten senior lecturers in both faculty of Dentistry and Pharmacy at International Medical University (IMU) and their feedback was incorporated into the revised questionnaire. In the second phase of piloting, the content validity of the questionnaire was pre-tested on 20 patients from the expected population to bring clarity and suitability for the local context.

This cross-sectional study involved a sample size of 270 individuals, the sample size was calculated based on the assumption that the prevalence of oral lesions in PLWHA is approximately 40% and by setting a standard error at 5% [6]. A simple random sampling technique proportionate to size was used to select the sample. A patients’ medical records number list (MRN) was obtained from the hospital and randomization was done based on the medical record numbers. The numbers identified were approached by the hospital administration to seek approval to participate. A name list of the patients agreeable to participate and scheduled for clinic visit during the period of the study was obtained. Name and identity card number of study participants were not taken to assure the confidentiality and anonymity of the patients. A written informed consent was obtained from each patient that agreed to participate in this study prior to the interview. Patients who were unable to give consent for reasons as, having communication barrier and with neurological or psychological problems were excluded from this study.

### Statistical analysis

Data were coded, entered and analyzed using Statistical Package for the Social Sciences, SPSS® version 18 with 0.05 as the level of significance.

Analysis of data involved two stages. The first stage was the descriptive statistics where frequencies, percentages and means were analyzed. The second stage involved using Chi-square to measure the association between demographics and Knowledge, practices, attitudes and previous training. Spearman rank correlation test to analyze the same variables for correlation.

## Results

### Demographics characteristics

Two hundred seventy patients were enrolled out of which 67% were males and 33% were females. The mean age of the patients was 39. Regarding the educational levels of the patients 27% were primary school graduates, 58% were secondary school graduates while 13% were university graduates. The demographics characteristics of patients are summarized in Table 1.

**Table 1:** Demographics of the participating individuals

	**Variables**	
**Gender**		**n = 270 (%)**
	Male	180 (67)
	Female	90 (33)
**Marital status**	Married	123 (46)
	Single	97 (36)
	Separated	44 (16)
	Divorced	6 (2)
	Widow	0
**Education Level**	Primary	75 (28)
	Secondary	160 (59)
	University	35 (13)
**Duration of HIV illness**	0-to 6 months	9 (3)
	6-12 months	22 (8)
	1-3 years	87 (32)
	4 years and above	152 (57)

### Knowledge, Attitude and Practices (KAP)

Upon implementation of the structured face to face interview and based on the patients responses, patients were grouped into knowledgeable (K) and non-knowledgeable (NK) regarding the oral manifestations of AIDS/HIV. Thirty seven % of patients were reported as knowledgeable while 63% of patients were non knowledgeable. Twenty four percent of male patients were knowledgeable and 76% were non-knowledgeable. Meanwhile 60% of female patients were reported as knowledgeable and 40% as non-knowledgeable. Results obtained showed no association or correlation between knowledge of the patients and their gender, age or marital status. Results also demonstrated that there is negative correlation between the level of knowledge of the patients and their level of education (R_s_ = −0.0029, P = 0.036). Table 2 represents demographics in relation to the knowledge of the patients.

**Table 2:** PLWHA’s knowledge of HIV/AIDS associated oral lesions

	**Variables**	**Knowledge**		**Chi-square**	**Correlation**
		**Knowledgeable**	**Non- Knowledgeable**		
		**n = 99(37%)**	**n = 171(63%)**		
**Gender**	Male	43 (24%)	137(76%)	P = 0.44	R_S_ = .049
					P = .423
	Female	54(60%)	36(40%)		
**Age**	mean age	37	39		R_S_ = -.104
					P=0.087
**Marital status**	Married	76(62%)	46(38%)	P = 0.842	R_S_ = −.015
					P = 0.802
	Single	89(64%)	49(36%)		
	Separated	3(75%)	1(25%)		
	Divorced	3(50%)	3(50%)		
	Widow	0	0		
**Education Level**	Primary	29 (39%)	46(61%)	P = 0.893	R_S_ = −.0029
					P = 0.036
	Secondary	58(36%)	102(64%)		
	University	12(34%)	23(66%)		
**Duration of HIV illness**	0-to 6 months	5 (56%)	4(45%)	P = 0.642	R_S_ = −.042
					P = 0.493
	6-12 months	9(41%)	13(59%)		
	1-3 years	31(36%)	56(64%)		
	4 years and above	54(36%)	98(64%)		

The responses to attitude items were grouped into positive and negative attitudes. Almost two thirds of the patients (64%) showed positive attitude while 36% of patients showed a negative attitude towards the AIDS associated oral lesions (Table 3). Seventy percent of the individuals who showed negative attitude towards oral lesions reported that cleaning the mouth of an HIV patient can increase the risk of transmission of the disease to the caregiver involved in cleaning of patient’s oral cavity.

**Table 3:** Attitudes of PLWHA towards HIV/AIDS associated oral lesions

	**Variables**	**Attitude**		**Chi-square**	**Correlation**
		**Positive n = 172(64%)**	**Negative n = 98(36%)**		
**Gender**	Male	110(61%)	70(39%)	P = 0.210	R_S_ = .076
					P = 0.212
	Female	62(69%)	28(31%)		
**Age**	(mean age)	39	38	P = 0.307	R_S_ = .044
					P = 0.474
**Marital status**	Married	78(64%)	44(36%)	P = 0.871	R_S_ = −.008
					P = 0.902
	Single	50(63%)	29(37%)		
	Separated	3(75%)	1(25%)		
	Divorced	3(50%)	3 (50%)		
	Widow	0	0		
**Education Level**	Primary	44(59%)	31(41%)	P = 0.415	R_S_ = .080
					P = 0.191
	Secondary	103(64%)	57(36%)		
	University	25(71%)	10(29%)		
**Duration of HIV illness**	0-to 6 months	7(77%)	2(23%)	P = 0.850	R_S_ = −.021
					P = 0.733
	6-12 months	14(64%)	8(36%)		
	1-3 years	55(63%)	32(37%)		
	4 years and above	96(63%)	56(37%)		

**Figure 1:**
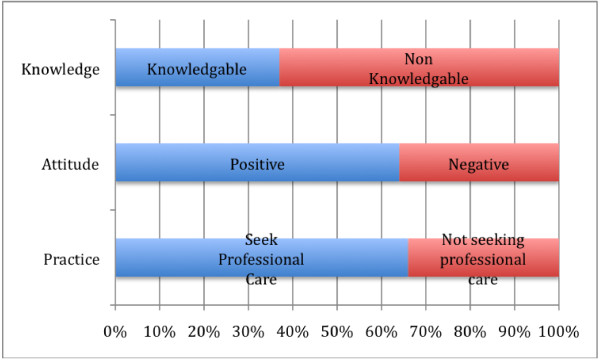
Knowledge, attitude and practices of HIV/Aids patients towards oral lesions.

Practices towards AIDS associated oral lesions were divided into seeking professional care and not seeking professional care when experiencing oral lesions. Two thirds of patients (66%) reported that they would seek professional care while 34% patients reported not seeking professional care. Results showed that there is no correlation between gender and practices. (R_s_ = 0.011, P = 0.857). Seeking professional care and related demographics are presented in (Table 4). Figure 1 represents the knowledge, attitudes and the practices of the patients towards AIDS/HIV associated oral lesions.

**Table 4:** Practices of PLWHA towards seeking professional care

	**Variables**	**Practices**		**Chi-square**	**Correlation**
		**Seeking professional care n = 178(66%)**	**Not seeking professional care n = 92(34%)**		
**Gender**	Male	118(66%)	62(34%)	P = 0.856	R_S_ = .011 P = .857
	Female	60(67%)	30(33%)		
**Age**	Mean age	38	39	P = 0.175	R_S_ = −.021 P = 0.732
**Marital status**	Married	79(65%)	43(35%)	P = 0.415	R_S_ = −.024 P = 0.695
	Single	92(67%)	46(33%)		
	Separated	4(100%)	0		
	Divorced	3(50%)	3(50%)		
	Widow	0	0		
**Education Level**	Primary	48(64%)	27(36%)	P = 0.214	R_S_ = −.026 P = 0.670
	Secondary	111(69%)	49(31%)		
	University	19(54%)	16(46%)		
**Duration of HIV illness**	0-to 6 months	4(44%)	5(56%)	P = 0.107	R_S_ = −.042 P = 0.490
	6-12 months	19(86%)	3(14%)		
	1-3 years	57(66%)	30(34%)		
	4 years and above	98(64%)	54(36%)		

### Effectiveness of training and demographics

Results revealed that 98% (n = 266) of the patients received training regarding their oral and general health. Effectiveness of this training was assessed and categorized into effective and non-effective training based on the patients’ perception. Ninety five percent of patients reported that their Training was effective while 5% of patients reported that the training received was not effective. Effectiveness of the training and the related demographics is shown in (Table 5).

**Table 5:** Reporting on effectiveness of training received by PLWHA

	**Variables**	**Training**		**Chi square n**	**Correlation**
		**Effective n = 252(95%)**	**Non-effective n = 14(5%)**		
**Gender**	Male	166(94%)	10(6%)	P = 0.669	R_S_ = −.026 P = 0.670
	Female	86(96%)	4(4%)		
**Age**	Mean age	38	40	P = 0.005	R_S_ = −.024 P = 0.691
**Marital status**	Married	113(94%)	7(6%)	P = 0.888	R_S_ = −.031 P = 0.610
	Single	129(95%)	7(5%)		
	Separated	4(100%)	0		
	Divorced	6(100%)	0		
	Widow	0	0		
**Education Level**	Primary	70(96%)	4(4%)	P = 0.338	R_S_ = −0.080 P = 0.191
	Secondary	149(94%)	10(6%)		
	University	33(100%)	0		
**Duration of HIV illness**	0-to 6 months	8(100%)	0	P = 0.659	R_S_ = −.071
	6-12 months	21(95%)	1(5%)		P = 0.250
	1-3 years	83(97%)	3(3%)		
	4 years and above	140(93%)	10(7%)		

### Knowledge Attitude Practices and Effectiveness of Training

Most of the patients felt that the training provided to them was effective. Statistical analysis showed no correlation or association between effectiveness of training and knowledge, attitude and practices of patients towards their oral lesions (Table 6 ).

**Table 6:** Effectiveness of training and knowledge attitude and practices

**Variables**		**Effective**	**Non-effective**	**Association**	**Correlation**
**Knowledge**	Knowledgeable	94(96%)	4(4%)	p = 0.516	R_2_ = .40 P = 0.512
	Non-Knowledgeable	158(94%)	10(6%)		
**Attitude**	Positive	160(95%)	9(5%)	p = 0.952	R_2_ = −.004 P = 0.952
	Negative	92(95%)	5(5%)		
**Practices**	Seek professional care	165(93%)	12(7%)	p = 0.118	R_2_ = −.096 P = 0.119
	Not Seeking professional care	87(98%)	2(2%)		

## Discussion

Within the population of the study it was found that majority of patients were males, similar findings were reported by the Ministry of Health in Malaysia as males represented 93% of total reported cases infected with HIV/AIDS [19]. Within the study the mean age of the population was found to be 39 which is supported by UNICEF reports on Malaysia which stated that the highest group of age which is infected by HIV is 30–39 years old [20]. Early identification of oral lesions in HIV/AIDS patient may ensure improving and maintaining quality of life and can predict HIV disease progression [21]. Hence, it is important to assess the patients’ knowledge, attitude and practices towards HIV/AIDS associated oral lesions.

Results related to the knowledge of HIV/AIDS associated oral lesions proved a negative correlation between level of education and patients’ knowledge. These findings were similar to those obtained in a study conducted in Tanzania to assess the awareness of PLWHAs towards oral manifestations of AIDS [22]. In another study conducted in Nigeria it was found that PLWHA exhibited fair knowledge towards the general manifestations of HIV/AIDS while poor knowledge was reported regarding the oral manifestations of the disease [23]. It is recommended to provide the PLWHA with the proper knowledge in regards to the HIV/AIDS associated oral lesions and oral health. The role of health care professionals should go beyond basic education and training and should extend to ensure that information is clearly presented to the patients with development of reinforcing educational programs.

Attitudes and beliefs of PLWHA towards oral care was assessed in this study and results revealed that more than half of the population exhibited a positive attitude towards the oral care. Within the literature it was found that there is several misconceptions related to the transmission of AIDS, these misconceptions are well established amongst the caregivers and is manifested as low willingness to help infected patients [24,25]. One of the common misconceptions is the idea that cleaning the mouth of an infected patient can increase the risk of transmission of the disease [12]. This study found that the same misconception is present amongst the PLWHA. Amongst the population that exhibited a negative attitude it was found that a high percentage of patients (70%) reported that cleaning of mouth of HIV patient can increase the risk of transmission of the disease to the person helping in the cleaning of oral cavity. The presence of these misconceptions amongst the patients themselves can be attributed to lack of adequate education related to various aspects of the disease and the absence of effective training of patients which can address the transmission of the disease. Those attitudes towards the disease might contribute to lower compliance to oral hygiene amongst PLWHA which was reported in literature as a result of several barriers including personal and family factors, dental fear, fear of stigmatism, lack of financial resources and giving dental care a low priority [26,27].

In this study, more than one third of the population did not include the option of seeking professional care in case of having oral manifestations; they have reported developing their own methods of handling the oral manifestations of the disease. Seeking professional care for oral lesions associated with HIV/AIDS is of great importance as the presence of oral lesions can have a significant impact on general well-being of individuals [28]. Hence, early diagnosis and management of these lesions plays a major role in decreasing the impact of the disease on the patient with subsequent less suffering and pain.

## Conclusion

In this study it was found that most of the patients were non-knowledgeable in relation to oral manifestations of the disease and that almost one third of the assessed population showed negative attitudes towards care of oral health and reported other options to handle oral lesions rather than seeking professional care. The gap in knowledge and the negative attitude in addition to the poor practices towards HIV associated oral lesion amongst the population is an alarming fact. Well-structured educational programs and innovative educational methodologies that can empower patients with knowledge and ensure that the patient has an access to the right information needed for decision-making should be developed.

### Limitations of the study

A larger population encompassing more regions is recommended. This study had smaller sample frame that may affect its generalization.

## Endnotes

^a^Sungai Buloh (National referral hospital of infectious diseases Kuala Lumpur-MALAYSIA

## Competing interests

The authors declare that they have no competing interests.

## Authors’ contributions

SAK and HO participated in concept, design, data collection and data analysis. JAY participated in data collections. SAK, JAY, HO and SSH participated in data interpretation and manuscript write up. All authors read and approved the final manuscript.

## Pre-publication history

The pre-publication history for this paper can be accessed here:

http://www.biomedcentral.com/1471-2458/12/850/prepub
